# Association Between Adolescent Blunt Use and the Uptake of Cigars

**DOI:** 10.1001/jamanetworkopen.2019.17001

**Published:** 2019-12-06

**Authors:** Janet Audrain-McGovern, Daniel Rodriguez, Emily Alexander, Stephen Pianin, Kymberle Landrum Sterling

**Affiliations:** 1 Department of Psychiatry, University of Pennsylvania Perelman School of Medicine, Philadelphia; 2 School of Nursing and Health Sciences, LaSalle University, Philadelphia, Pennsylvania; 3 School of Public Health, University of Texas Health Sciences Center, Dallas

## Abstract

**Importance:**

Almost two-thirds of adolescents who smoke blunts also report using cigars. Although adolescent co-use of blunts and cigars is prevalent, whether adolescent blunt use is associated with progression to current cigar use (past 30-day use) and increased frequency of use is unknown.

**Objective:**

To determine whether adolescent use of blunts is associated with progression to cigar use (past 30-day use) and increased number of days used (in the past 30 days) across the subsequent 24 months compared with adolescents who have never used blunts.

**Design, Setting, and Participants:**

In this prospective cohort study of adolescents from 4 public high schools outside of Philadelphia, Pennsylvania, in-classroom surveys were administered at baseline (fall 2016, beginning of 9th grade) and at 6-month intervals for the following 24 months (fall 2018, beginning of 11th grade). Data analyses were conducted in September and October 2019.

**Exposures:**

Self-report of lifetime (ever) blunt use at baseline (age 14 years).

**Main Outcomes and Measures:**

Progression to current cigar use (use in the past 30 days) and escalation in the frequency of use (number of days used in the past 30 days) across the subsequent 24 months.

**Results:**

Of 1825 participants, 907 (49.7%) were female, 1330 (72.9%) were white, and 376 (20.6%) were Hispanic; the mean (SD) age at baseline was 14.38 (0.55) years. In all, 257 participants (14.0%) reported ever blunt use. Mixed-effects models revealed that ever blunt use at baseline vs never blunt use was associated with progression to current cigar use (past 30 days use: odds ratio, 22.66; 95% CI, 11.34-45.27) but not escalation in the number of days used across the following 24 months (β = 0.13; 95% CI, −0.17 to 0.43).

**Conclusions and Relevance:**

These findings highlight the risk that blunt use may pose for subsequent cigar use among adolescents. Policies and public health campaigns addressing marijuana as well as cigars will be important to reduce adolescent blunt use and cigar use.

## Key Points

**Question:**

Is blunt use among adolescents associated with progressing to current cigar use and more frequent cigar use compared with adolescents who have never used blunts?

**Findings:**

In this cohort study of 1825 US adolescents, ever vs never blunt use at age 14 years was significantly associated with progression to current cigar use across the following 24 months. Blunt use was not significantly associated with the number of days of cigar use across follow-up.

**Meaning:**

These findings highlight the risk that blunt use may pose for subsequent cigar use among adolescents.

## Teaser

This cohort study examines the association of blunt use with cigar use among US adolescents.

## Introduction

Blunting a cigar is a popular form of marijuana use among adolescents.^1^ Blunting, or making a blunt, is a process whereby some or all of the tobacco is removed from a cigar product (eg, large cigar, little cigar, cigarillo, or tobacco rolling paper), then replaced with marijuana and smoked.^2,3,4^ Estimates suggest that 12% to 19% of adolescents have smoked a blunt in the past month.^5,6^ Almost two-thirds of adolescents who smoke blunts also report using cigars.^5^ Indeed, 40% of adolescents who have ever used a little cigar or cigarillo did so to modify it for blunt smoking.^7^

More severe and persistent negative outcomes, such as addiction, have been documented when combustible tobacco and marijuana use is initiated during adolescence, especially when both substances are regularly used.^8,9,10^ Adolescents who modify cigars for blunt use are exposed to nicotine through the tobacco wrap despite removal of the tobacco.^11,12^ The coadministration of nicotine and cannabis through blunting may increase the odds that an adolescent will become dependent on both nicotine and cannabis.^2^ As nicotine dependence emerges, adolescents may begin using cigars as purchased and not just for blunting.

Cross-sectional research has documented the co-use of blunts and cigars among adolescents^5,7^; however, to our knowledge, whether blunt use is associated with cigar uptake and escalation in the frequency of cigar use has yet to be investigated. This prospective cohort survey of adolescents in high school sought to determine whether adolescents who ever used blunts, compared with those who did not, were more likely to become current (past 30-day) cigar users and to progress to more frequent use across the following 24 months. Evidence documenting that the use of blunts is associated with cigar use would help guide marijuana- and cigar-related policies to protect adolescents.

## Methods

### Participants and Procedures

Participants were adolescents in the ninth grade taking part in a longitudinal cohort study of the associations among combustible cigarette smoking, electronic cigarette use, and other tobacco use. Participants were enrolled in 1 of 4 public high schools in suburban Philadelphia, Pennsylvania. The schools were selected such that our sample would be demographically representative of adolescents nationwide in terms of sex, race, ethnicity, and annual household income. The cohort participants were drawn from 2198 students identified through class rosters at the start of ninth grade. Adolescents were ineligible to participate if they had a severe learning disability or if they did not speak fluent English. Based on the selection criteria, a total of 2017 of the 2198 students (92%) were eligible to participate.

Parents were mailed a study information letter (active information) with a telephone number to call to obtain answers to any questions and to decline consent for their adolescent to participate (passive consent). Of the 2017 eligible adolescents, 17 (1%) had a parent who actively declined their adolescent’s participation. Adolescents with parental consent were approached to provide their written assent for study participation. The 124 adolescents (6%) who were absent on the assent/baseline survey days and the 41 adolescents (2%) who did not provide assent owing to lack of interest were not enrolled in the cohort. Thus, 1835 of the 2000 adolescents with consent (92%) provided their assent to participate and completed a 40-minute paper-and-pencil survey. This baseline, or wave 1, survey was completed on site during compulsory classes in the fall of 2016.

Adolescents completed 4 paper-and-pencil follow-up surveys at 6-month intervals, with 1687 (92%) completing a survey at wave 2 (spring 2017), 1658 (90%) completing a survey at wave 3 (fall 2017), 1643 (89%) completing a survey at wave 4 (spring 2018), and 1601 (87%) completing a survey at wave 5 (fall 2018). Adolescents were assigned a unique identifier at the baseline survey. A cover page with the adolescent’s name connected to their identifier on the follow-up survey was removed at the time of survey receipt. The participants included in this study are 1825 adolescents who had complete data on the study variables at baseline. Missingness at subsequent waves was largely due to loss to follow-up (ie, adolescent moved and could not be reached). The only variable associated with loss to follow-up was non-Hispanic ethnicity (odds ratio [OR], 1.92; 95% CI, 1.34-2.75). The institutional review board of the University of Pennsylvania and the administration of each of the 4 high schools approved the study. Reporting of the results follows the Strengthening the Reporting of Observational Studies in Epidemiology (STROBE) reporting guideline. Data analyses were conducted in September and October 2019.

### Measures

#### Cigar Use

In a series of frequently used survey questions, adolescents were asked whether they had ever used large cigars, little cigars, or cigarillos and whether they had used one of these types of cigars in the past 30 days.^13^ Those who used cigars in the past 30 days were asked how many days in the past 30 days cigars were used.^13^ To aid accurate reporting, brand examples were provided.^14,15,16^ As is standard, current use was defined as using a cigar on at least 1 day in the past 30 days.^5,13^ Escalation in cigar use among those adolescents who reported current use (past 30 days) at baseline was defined as an increase in the number of days in the past 30 days in which a cigar was used compared with baseline. Cigar use was measured at all 5 waves.

#### Covariates

Demographic characteristics such as sex, race, and ethnicity were assessed using self-report items. These demographic variables were included in the model to characterize the sample. Similar to the assessment of cigar use, adolescents were asked whether they had ever used a blunt, whether they had used a blunt in the past 30 days, and on how many days in the past 30 days blunts were used.^17^ To aid accurate reporting, blunts were defined as marijuana rolled in cigar or tobacco wraps.^5^ We included sensation seeking and combustible cigarette smoking in the model to control for its potentially overlapping risk for cigar and blunt use.^18,19,20,21^ Sensation seeking was measured with the 8-item Brief Sensation Seeking Scale (with 0 indicating strongly disagree to 4 indicating strongly agree, scores ranging from 0-32, and higher scores indicating greater sensation seeking).^22^ Lifetime combustible cigarette smoking was assessed by asking adolescents whether they had ever tried smoking a cigarette (even a few puffs).^13,16^

### Statistical Analysis

Mixed-effects models were used to evaluate the association between ever blunt use at baseline and (1) progression to current (past 30-day) cigar use and (2) escalation in the number of days cigars were used in the past 30 days across the subsequent 24 months. A logistic regression model was used to assess the association of ever blunt use at baseline with repeated measures of past 30-day cigar use (yes vs no) across waves 2 through 5. A binary mixed-effects model with a logit link and random intercept was fit to the data.

General linear mixed modeling was used to assess escalation in the number of days of cigar use in the past 30 days among adolescents who reported cigar use in the past 30 days. For this model, we fit a log-normal mixed-effects model including a random intercept. Unadjusted models were followed by models including the covariates. Cigar use was included in the models at wave 1 to control for any previous use and to assess the prospective association between blunt use and the progression to current (past 30-day) cigar use and escalation in the number of days cigars were used in the past 30 days across waves 2 through 5.

Means and standard deviations were used to describe continuous variables. Frequency distributions and proportions were used to describe categorical variables. Statistical significance was set at 2-tailed *P* < .05. Both models were run using the GLIMMIX procedure in SAS software version 9.4 (SAS Institute). Supplementary analyses were conducted to examine an alternative association between blunt use and cigar use, that is, whether cigar use was associated with the uptake of blunt use among adolescents.

## Results

### Study Sample

Of 1825 participants, 907 (49.7%) were female, 1330 (72.9%) were white, and 376 (20.6%) were Hispanic; the mean (SD) age at baseline (wave 1) was 14.38 (0.55) years. The sample characteristics are presented in Table 1. At baseline, 257 participants (14.0%) reported ever using a blunt and 159 (8.7%) reported ever using a cigar. The mean (SD) number of days that blunts were used in the past 30 days was 8.71 (9.54) at wave 1 and 9.29 (9.11) at wave 5. The mean (SD) number of days that cigars were used in the past 30 days was 7.78 (8.65) at wave 1 and 8.09 (8.13) at wave 5. The mean (SD) sensation-seeking score was 13.94 (7.13).

**Table 1.** Sample Participant Characteristics

Variable	No. (%)
Sex	
Male	918 (50.3)
Female	907 (49.7)
Race	
White	1330 (72.9)
Black	261 (14.3)
Other	234 (12.8)
Ethnicity	
Hispanic	376 (20.6)
Not Hispanic	1449 (79.4)
Cigarette smoking	
Never	1716 (93.26)
Ever	124 (6.74)
Baseline cigar use	
Never	1677 (91.34)
Ever	159 (8.66)
Baseline blunt use	
Never	1583 (86.03)
Ever	257 (13.97)

### Association Between Blunt Use and Subsequent Cigar Use

Table 2 presents the associations between ever blunt use and progression to current cigar use (30-day cigar use) across waves 2 through 5. Ever blunt use at baseline was associated with a more than 20-fold increase in the odds of progressing to current cigar smoking over the 5-wave period (odds ratio [OR], 22.66; 95% CI, 11.34-45.27). Several other covariates had significant associations with progression to 30-day cigar use across the 5 waves. Ever smoking a combustible cigarette at baseline was associated with a 15-fold increase in the odds of progressing to 30-day cigar use (OR, 15.12; 95% CI, 6.25-36.59). Finally, each 1-SD increase in sensation seeking was associated with a 10% increase in the odds of 30-day cigar use (OR, 1.10; 95% CI, 1.05-1.15).

**Table 2.** Association Between Adolescent Ever Blunt Use and Progression to Current (Past 30-Day) Cigar Use^a^

Baseline Variable	Odds Ratio (95% CI)
Time^b^	1.08 (0.97-1.21)
Blunts	22.66 (11.34-45.27)
Sex	0.91 (0.50-1.66)
Race^c^	
Black	0.43 (0.16-1.11)
Other	0.70 (0.26-1.88)
Ethnicity	0.97 (0.47-2.00)
Combustible cigarettes	15.12 (6.25-36.59)
Sensation seeking	1.10 (1.05-1.15)

^a^ Blunt use was categorized as 0 for never used and 1 for ever used; combustible cigarette use as 0 for never smoked and 1 for ever smoked; sex as 0 for male and 1 for female; and ethnicity as 0 for Hispanic and 1 for not Hispanic.

^b^ Time unit is 6 months.

^c^ Race was dummy coded, with white race being the comparison category. Black race was categorized as 0 for no and 1 for yes; other race as 0 for no and 1 for yes.

Ever blunt use was not associated with an escalation in the frequency of cigar use (number of days that cigars were used in the past 30 days) across the following 24 months (β = 0.13; 95% CI, −0.17 to 0.43) (Table 3). Only sex had a significant association with the frequency of cigar use. Being female was associated with a 26% decrease (β = −0.2639; 95% CI, −0.529 to −0.001) in the number of days of cigars use.

**Table 3.** Association Between Adolescent Ever Blunt Use and Escalation in Cigar Use Frequency (Number of Days in Past 30 Days)^a^

Baseline Variable	β (95% CI)
Intercept	1.65 (1.08-2.22)
Time^b^	−0.02 (−0.10 to 0.07)
Blunts	0.13 (−0.17 to 0.43)
Sex	−0.26 (−0.53 to 0.001)
Race^c^	
Black	0.26 (−0.16 to 0.67)
Other	−0.39 (−0.88 to 0.1)
Ethnicity	−0.17 (−0.48 to 0.14)
Combustible cigarettes	0.08 (−0.20 to 0.36)
Sensation seeking	0.002 (−0.02 to 0.02)

^a^ Blunt use was categorized as 0 for never used and 1 for ever used; combustible cigarette use as 0 for never smoked and 1 for ever smoked; sex as 0 for male and 1 for female; and ethnicity as 0 for Hispanic and 1 for not Hispanic.

^b^ Time unit is 6 months.

^c^ Race was dummy coded, with white race being the comparison category. Black race was categorized as 0 for no and 1 for yes; other race as 0 for no and 1 for yes.

### Association Between Cigar Use and Subsequent Blunt Use

In a similar analytic fashion, we evaluated the association between ever cigar use at baseline, progression to current blunt use, and escalation in the number of days blunts were used in the past 30 days across the subsequent 24 months.^23,24^ Current blunt use was defined as using blunts on at least 1 day in the past 30 days.^5,13,17^ Escalation in the frequency of blunt use among those adolescents who reported current use (past 30 days) at baseline was defined as an increase in the number of days in the past 30 days in which a blunt was used. Blunt use was included in the models at wave 1 to control for any previous use and to assess the prospective association between cigar use and the progression to current (past 30-day) blunt use as well as escalation in the number of days blunts were used in the past 30 days across waves 2 to 5.

Table 4 presents the associations between ever cigar use and progression to current blunt use (30-day blunt use) across waves 2 to 5. Ever smoking a cigar was associated with a 14-fold increase in the odds of progressing to 30-day blunt use (OR, 14.25; 95% CI, 6.22-32.63). Every 6-month passage of time was associated with a 39% increase in the odds of using a blunt in the past 30 days (OR, 1.39; 95% CI, 1.28-1.50). Ever smoking a combustible cigarette (OR, 8.87; 95% CI, 3.64-21.64) and sensation seeking (OR, 1.13; 95% CI, 1.10-1.17) were also associated with progressing to 30-day blunt use. By contrast, being of non-Hispanic ethnicity was associated with decreased odds of 30-day blunt use (OR, 0.47; 95% CI, 0.28-0.78).

**Table 4.** Association Between Adolescent Ever Cigar Use and Progression to Current (Past 30-Day) Blunt Use^a^

Baseline Variable	Odds Ratio (95% CI)
Time^b^	1.39 (1.28-1.5)
Cigar use	14.25 (6.22-32.63)
Sex	1.02 (0.67-1.55)
Race^c^	
Black	1.21 (0.67- 2.2)
Other	0.52 (0.26-1.03)
Ethnicity	0.47 (0.28-0.78)
Combustible cigarettes	8.87 (3.64-21.64)
Sensation seeking	1.13 (1.10-1.17)

^a^ Blunt use was categorized as 0 for never used and 1 for ever used; combustible cigarette use as 0 for never smoked and 1 for ever smoked; sex as 0 for male and 1 for female; and ethnicity as 0 for Hispanic and 1 for not Hispanic.

^b^ Time unit is 6 months.

^c^ Race was dummy coded, with white race being the comparison category. Black race was categorized as 0 for no and 1 for yes; other race as 0 for no and 1 for yes.

As shown in Table 5, every 6-month passage of time was associated with an 8% increase in the number of days blunts were used in the past 30 days (β = 0.08; 95% CI, 0.02-0.13). Ever smoking a cigar was associated with a 38% increase in the number of days of blunt use in the past 30 days (β = 0.38; 95% CI, 0.10-0.65). Being female was associated with a 45% decrease in the number of days of blunt use in the past 30 days (β = −0.45; 95% CI, −0.65 to −0.25).

**Table 5.** Association Between Adolescent Ever Cigar Use and Escalation in Blunt Use Frequency (Number of Days in Past 30 Days)^a^

Baseline Variable	*b* (95% CI)
Intercept	1.40 (1.01-1.78)
Time^b^	0.08 (0.02 to 0.13)
Cigar use	0.38 (0.10 to 0.65)
Sex	−0.45 (−0.65 to −0.25)
Race^c^	
Black	0.12 (−0.16 to 0.40)
Other	−0.17 (−0.53 to 0.19)
Ethnicity	−0.06 (−0.29 to 0.17)
Combustible cigarettes	0.19 (−0.10 to 0.47)
Sensation seeking	−0.003 (−0.02 to 0.01)

^a^ Blunt use was categorized as 0 for never used and 1 for ever used; combustible cigarette use as 0 for never smoked and 1 for ever smoked; sex as 0 for male and 1 for female; and ethnicity as 0 for Hispanic and 1 for not Hispanic.

^b^ Time unit is 6 months.

^c^ Race was dummy coded, with white race being the comparison category. Black race was categorized as 0 for no and 1 for yes; other race as 0 for no and 1 for yes.

## Discussion

The current study offers new evidence for a prospective association between adolescent blunt use and the use of cigars. Adolescents who had ever used a blunt at age 14 years had more than a 20-fold increase in the odds of becoming a current cigar user across the 24-month follow-up compared with adolescents who had never used a blunt. Whereas previous research has documented an increased vulnerability to marijuana use associated with newer forms of tobacco (eg, electronic cigarettes) among adolescents,^16^ the present findings highlight the risk that marijuana use via blunts poses for cigar uptake.

Supplementary analyses revealed that adolescents who had ever used a cigar at age 14 years had a 14-fold increase in the odds of progressing to 30-day blunt use, and a 38% increase in the frequency of blunt use across the subsequent 24 months. Coupled with our findings that blunt use at age 14 years was associated with cigar uptake, we can speculate that youth who initiate blunt use may subsequently begin using cigars as intended and that cigar users may subsequently begin to modify their cigars for blunt use. Indeed, using data from the US National Surveys on Drug Use and Health, a recent study^25^ observed that approximately half of young people who reported having smoked a blunt reported no prior cigar use, while the other half reported that cigar use preceded the initiation of blunt use.

Blunt use and cigar use among adolescents are likely fostered through misperceptions regarding the health risks associated with use. Adolescents perceive cigars as less harmful than cigarettes, despite similar health consequences.^26^ Adolescents perceive blunt use as more socially acceptable, less harmful to their health, and less addictive than cigarettes.^27^ Lower risk perceptions associated with marijuana use are accompanied by more perceived benefits.^28^ Nationwide, the percentage of adolescents who perceive that marijuana use is associated with significant risks has decreased from 30.9% to 21.4% among 10th graders from 2010 to 2018.^29^ These decreases may reflect the evolving regulatory landscape—as of 2018, 33 states and the District of Columbia had legalized marijuana in some form.^30^ Educating adolescents about the health consequences associated with blunt smoking, cigar smoking, and the risk for becoming a regular cigar and blunt user seems warranted.

Furthermore, some cigars, especially cigarillos, are specifically marketed to promote their use in blunt making. These include features that facilitate blunt making, such as perforations in the tobacco wrap to increase the ease with which the tobacco can be removed and marijuana can be added.^31^ In addition, cigarillos are often sold in a foil pouch, which is popular for storing marijuana.^31^ Whereas these marketing practices are directed toward those who use cigars for blunt making, they may ultimately benefit cigar manufacturers as adolescents become nicotine dependent and begin using cigars as intended, not just for blunting.

Given that nicotine prolongs and heightens the reinforcing effects of marijuana,^20,32,33^ it is not surprising that coadministering nicotine and cannabis through blunts has been implicated in persistent use of both substances.^2,34^ Flavored little cigar and cigarillo use has been tied to blunt smoking,^35^ as young adults report that flavoring enhances the taste of the marijuana.^36^ As such, flavoring may further add to the abuse liability and likelihood of persistent use. In November 2018, the US Food and Drug Administration Center for Tobacco Products announced plans to restrict the sale of flavored cigars that were on the market on August 8, 2016. Although this constituted a positive step to reducing exposure to some flavored cigars, those on the market prior to February 15, 2007, are to be “grandfathered” by the Food and Drug Administration and will still be available for purchase. Consequently, this regulatory policy, along with the spread of marijuana legalization in the United States, may inadvertently contribute to continued cigar use and cigar-blunt use among youth.

### Strengths and Limitations

As the first study, to our knowledge, to examine the longitudinal association between adolescent blunt use and adolescent cigar use, this study has strengths as well as limitations. The sample was demographically diverse and adolescents were measured during a developmentally vulnerable period for tobacco use. We also used repeated measures of cigar use and blunt use, analyzing the data in a longitudinal fashion across 5 points spanning 2 years. In addition, we controlled for several variables that could account for an association between adolescent cigar use and blunt use. Even after controlling for these variables, the model showed an association between adolescent use of cigars and blunts. However, it is important to note that the present study did not find an association between blunt use and escalation in the frequency of cigar use. Progression to significantly more frequent use may unfold across a longer period of time.

A potential limitation of this study is that we did not measure how blunts were made. While blunt use and cigar use were assessed as 2 separate behaviors, it is unclear whether blunts were made with little cigars, large cigars, cigarillos, or tobacco leaf wraps. Relatedly, we did not measure preference for flavored cigars and its association with blunt use. To offer increasing clarity into the association between adolescent blunt use and subsequent cigar use, future research will need to delineate specific features of blunt making and cigar use among adolescents. In addition, given that our overall marijuana use item assessed use from all sources, including blunts, we were not able to include it in the model and control for other sources of marijuana use.

## Conclusions

The findings of this cohort study highlight the risk that blunt use may pose for subsequent cigar use among adolescents. In addition, the findings point to the risk that cigar use may pose for subsequent and escalating blunt use among adolescents. This is concerning, as co-use of tobacco and marijuana through blunting may present an additive risk for toxicant and carcinogen exposure.^37^ Policies and public health campaigns addressing marijuana as well as cigars will be important to reduce adolescent blunt use and cigar use.
