# Error in Table Notes

**DOI:** 10.1001/jamanetworkopen.2019.19372

**Published:** 2019-12-18

In the Original Investigation titled “Association Between Adolescent Blunt Use and the Uptake of Cigars,”^1^ published December 6, 2019, there was an error in footnote a of Table 4 and Table 5. Footnote a for both tables should have read “Cigar use was categorized as 0 for never used and 1 for ever used…” instead of “Blunt use was categorized as 0 for never and 1 for ever used...” This article has been corrected.^1^
